# Association of vitamin D and bisphenol A levels with cardiovascular risk in an elderly Italian population: results from the InCHIANTI study

**DOI:** 10.1007/s11357-024-01193-1

**Published:** 2024-06-05

**Authors:** Maria Luisa Brandi, Francesca Marini, Simone Parri, Stefania Bandinelli, Teresa Iantomasi, Francesca Giusti, Eleonora Talluri, Giovanna Sini, Fabrizio Nannipieri, Santina Battaglia, Giovanni Tripepi, Colin Gerard Egan, Luigi Ferrucci

**Affiliations:** 1 Fondazione FIRMO Onlus (Fondazione Italiana Ricerca Sulle Malattie Dell’Osso), F.I.R.M.O. Foundation, Via San Gallo, 123, 50129 Florence, Italy; 2 Geriatric Unit, Azienda Sanitaria Toscana Centro, Florence, Italy; 3 Department of Experimental and Clinical Biomedical Sciences, University of Florence, Florence, Italy; 4 Medical Research, Abiogen Pharma, Pisa, Italy; 5 National Research Council (CNR), Ospedali Riuniti, Reggio Calabria, Italy; 6 CE Medical Writing SRLS, Pisa, Italy; 7 Longitudinal Study Section, Translation Gerontology Branch, National Institute On Aging, Baltimore, MD USA; 8 Donatello Bone Clinic, Villa Donatello Hospital, Sesto Fiorentino, Italy

**Keywords:** Bisphenol A, Cardiovascular risk, Vitamin D, 25(OH)D, 1,25(OH)_2_D, Elderly people

## Abstract

Few studies have evaluated the association between circulating levels of 25-hydroxyvitamin D (25(OH)D), 1,25-dihydroxyvitamin D (1,25(OH)_2_D), and the endocrine disruptor bisphenol A (BPA), with risk of cardiovascular (CV) disease in elderly individuals. This was a cross-sectional study in a subgroup of elderly people from the InCHIANTI Biobank in Italy. We examined the association between circulating serum vitamin D metabolites, 1,25(OH)_2_D, 25(OH)D, and the endocrine disrupting agent BPA, with an arbitrary CV risk score and the European Society of Cardiology-based 10-year CV risk (SCORE2/SCORE2-OP) using univariate and multiple regression. In 299 individuals, blood samples were tested for serum values of 25(OH)D, 1,25(OH)_2_D and urinary BPA levels. One hundred eighty individuals (60.2%) were deficient (< 20 ng/ml) in 25(OH)D. Levels of 25(OH)D and 1,25(OH)_2_D were negatively correlated with CV risk score (*p* < 0.0001 for both) as well as SCORE2/SCORE2-OP (*p* < 0.0001 for both) while BPA levels were positively correlated with both CV risk scores (*p* < 0.0001 for both). In a logistic regression model, male gender (odds ratio; OR: 2.1, 95% CI:1.1–3.8, *p* = 0.022), obesity (OR:2.8, 95% CI:1.2–6.5, *p* = 0.016) and BPA levels ≥ 110 ng/dl (OR:20.9, 95% CI:9.4–46.8, *p* < 0.0001) were associated with deficient levels of 25(OH)D. 1,25(OH)2D levels < 41 ng/dl and 25(OH)D levels < 20 ng/ml were associated with CV risk score ≥ 3 (OR: 4.16, 95% CI: 2.32–7.4, p < 0.0001 and OR: 1.86, 95% CI: 1.02–3.39, p = 0.044) respectively and 1,25(OH)2D levels < 41 ng/dl were associated with SCORE2/SCORE2-OP of ≥ 20% (OR:2.98, 95% CI: 1.7–5.2, p = 0.0001). In this cross-sectional analysis, BPA exposure was associated with significantly reduced levels of vitamin D that in turn were significantly associated with increased CV risk.

**Supplementary Information:**

The online version contains supplementary material available at 10.1007/s11357-024-01193-1.

## Introduction

Vitamin D is an essential regulator of calcium homeostasis [1] and it is well recognised that normal levels of vitamin D can prevent and cure bone diseases, such as rickets osteomalacia, and osteoporosis [2, 3]. In addition, to its primary role in calcium and bone metabolism, vitamin D has been shown to exert other biological roles, such as reduction of inflammation, modulation of cell growth, regulation of immune and neuromuscular function and of glucose metabolism [4]. A chronic deficiency of vitamin D can predispose and/or facilitate the development of many diseases in humans.

Vitamin D status is assessed by serum values of the transport form of vitamin D, the 25-hydroxyvitamin D (25(OH)D), and according to the Endocrine Society [2], vitamin D deficiency is defined as circulating levels of 25(OH)D less than 20 ng/ml and insufficiency of 25(OH)D between 20 and 29 ng/ml. Vitamin D deficiency or insufficiency are common conditions worldwide, occurring in 30–50% of the global population [5, 6], across all geographical, ethnical and age groups, and particularly in the elderly people [7, 8] because of poor exposure to sunlight, reduced skin synthesis capacity, and reduced intestinal absorption [9, 10]. In addition, vitamin D deficiency is also seen in elderly individuals without these risk factors [11].

Results from recent observational findings and Nonlinear Mendelian analysis indicate that an insufficient or deficient vitamin D status may increase the risk of cardiovascular diseases (CVD), with an inverse nonlinear association between vitamin D status and the occurrence of CVD events and mortality [12, 13].

Bisphenol A (BPA) is a chemical organic compound primarily used in the manufacture of polycarbonate (PC) raw material plastic that is widely used in personal care and consumer products [14]. At sufficient levels, BPA can mimic the action of oestrogen and may lead to endocrine dysfunction in humans [15], such as disorders of the reproductive system, obesity and diabetes, and increase the risk of cancer [16], hence recognised as "endocrine disruptor chemicals" (EDCs). Epidemiological studies have shown that higher urinary BPA concentrations are associated with various types of CVDs, including hypertension, angina, heart attack and coronary and peripheral arterial disease [17, 18]. In a recent study, retrospectively performed on a population-based NHANES database (2011–2016) on 1,467 individuals, higher concentrations of BPA were found to be associated with a higher risk for CVDs [19].

In a previous cross-sectional study, performed by our Research Group on a subpopulation of 299 individuals from the InCHIANTI database, we observed a strong inverse relationship between urinary BPA concentrations and circulating vitamin D levels [20]. Indeed, it has also been recently demonstrated elsewhere that exposure to BPA and other EDCs (e.g. phthalates) can be associated with a reduction in vitamin D levels [21, 22].

In this post-hoc analysis, we explored the same InCHIANTI subpopulation to evaluate the association between circulating levels of 25(OH)D and 1,25(OH)_2_D, and urinary BPA with cardiovascular (CV) risk.

## Methods

### Study design

The InCHIANTI (“Invecchiare in Chianti”, aging in the Chianti area) study was originally designed in 1998 to evaluate factors contributing to the decline of mobility in later life, by performing longitudinal medical examinations, questionnaires and related functional tests on the elderly population of two small towns located in the Chianti Region, Greve in Chianti and Bagno a Ripoli, Italy [23], through baseline (1998–2000) and follow-up visits performed every three years from 1998 to 2014.

The present non-interventional monocentric cross-sectional study included a subpopulation of 299 individuals from the InCHIANTI database and it was conducted in full compliance with Italian legislation regarding non-interventional studies and the principles of the Declaration of Helsinki. All participants provided written informed consent for the anonymous use of their clinical and biochemical data according with the European Legislative Decree 2016/679. The Regional Ethics Committee for Clinical Trials of the Tuscany Region (Section, Area Vasta Centro; Careggi, Firenze) approved this study (protocol number: 13929_bio) on 08/07/2019, which was conducted according to the regulations for observational studies [24].

### Study population and sample collection

We retrospectively retrieved available clinical, biochemical and therapeutic data from the InCHIANTI database and obtained blood and urine samples stored in the InCHIANTI Biobank from 299 individuals (165 women and 134 men), aged 72.8 ± 15.7 years, collected during the fourth follow-up (from June 2013 to July 2014).

Intravenous blood samples were initially collected after an overnight 8 h fasting from the InCHIANTI population, delivered to the laboratory within 2 h for the retrieval of serum and plasma that was then stored, in 0.3 ml aliquots, at −80 °C in the InCHIANTI Biobank, at the Piero Palagi Hospital, Florence, prior to undertaking biochemical assays. Urine samples were collected first in the morning after an over-night 8 h fasting and then stored at −80 °C InCHIANTI Biobank until further use.

### Laboratory assays

Assays for 25(OH)D, 1,25(OH)_2_D, parathyroid hormone (PTH), and creatinine were performed at the central laboratory of the Azienda Ospedaliero-Universitaria Careggi (AOUC), Florence, Italy. Measurement of BPA and vitamin D binding protein (VDBP) were performed at the laboratory of the Fondazione FIRMO Onlus (FirmoLab), Florence, Italy. While serum values of calcium, magnesium, total cholesterol, LDL-cholesterol, HDL-cholesterol, triglycerides and glucose were retrospectively retrieved from the fourth follow-up in the InCHIANTI database.

Serum 1,25(OH)_2_D and 25(OH)D levels were measured by radioimmunoassay (Liason, DiaSorin, Saluggia, Italy). Serum PTH was analysed using a sandwich electrochemiluminescence immunoassay (ECLIA) by Elecsys 2010 Modular Analytics E170 COBAS 602 (Roche Diagnostics GmbH, Mannheim, Germany). Serum creatinine was measured by an enzymatic method with calibration traceable to a reference procedure (isotope dilution-mass spectrometry) and automatized on a Cobas 8000. Serum VDBP was measured by Enzyme-Linked Immuno Assay method (R&D Systems). BPA analysis was performed on urine samples using the HPLC/Mass Spectrometry using solid phase extraction coupled with high performance isotope dilution tandem liquid chromatography-mass spectrometry with focus peak. A comprehensive quality control system was implemented to prevent samples from being contaminated during handling, storage and analysis. For BPA concentrations below the detection level (116/1455 [8%]), a value of 0.3 ng/mL (30 ng/dl) was assigned in the NHANES survey and this value was used in the present analysis [25].

### Estimate of CV risk

To explore the potential association between vitamin D metabolites and BPA with CV risk we used two independent scores, an arbitrary measure of CV risk and the SCORE2/SCORE2-OP proposed by the European Society for Cardiology (ESC).

In the arbitrary measure of CV risk, that has been previously described elsewhere [26–28], a total of 8 different clinical variables or risk factors were considered and stratified into high/low or absence/presence yielding a maximum score of 8, representing highest possible CV risk. For continuous variables; age and BMI, cut off-values were ≥ 75 years and ≥ 30 kg/m^2^ respectively. The remaining dichotomous variables (absence or presence in the previous 3 years) included smoking, dyslipidaemia, diabetes, hypertension and a CV event (angina pectoris or peripheral arterial disease or myocardial infarction or stroke). Male gender was also included as a dichotomous CV risk variable.

We also used the 10-year CV risk based on the SCORE2/SCORE2-OP model proposed by the 2021 ESC Guidelines on Cardiovascular Disease Prevention [29, 30]. The individual 10-year risk of a CV event (expressed as %), was estimated according to SCORE2 (as calibrated for moderate risk European regions) and its equivalent for patients older than 70 years (SCORE2-OP) based on the variables age, sex, smoking, systolic blood pressure (SBP), total cholesterol and high-density lipoprotein (HDL) cholesterol. In our database, systolic blood pressure values were not available. However, information on hypertensive status was available and this allowed us to assign a score of 140 mm/Hg for hypertension and 120 mm/Hg to those without hypertension that are recognised cut-off values defining normal and hypertensive individuals according to International guidelines [31, 32].

### Statistical analysis

Continuous quantitative variables were summarized using either mean ± standard deviation (SD) or median and range. Categorical variables were expressed as numbers and percentages. Normality of the data distribution was assessed using the Kolmogorov–Smirnov test and by examining asymmetry and kurtosis values. For normally distributed continuous variables, comparisons were performed using the Student's t-test while proportions for categorical variables were compared using either the χ^**2**^ test or Fisher's exact test. Univariate correlations were assessed using the Pearson’s correlation coefficient and summarised in a correlation matrix. Urinary and serum laboratory variables were log transformed (as they were not distributed normally) prior to univariate and multivariate analyses. Multiple logistic regression models were used to assess the strength of the association between individuals having deficient levels of 25(OH)D (i.e. < 20 ng/mL; as dependent variable) and a range of clinical and laboratory variables (age < 75 vs. ≥ 75 years, gender, BMI < 30 vs. ≥ 30 kg/m^2^, diabetes (yes vs. no), dyslipidaemia (yes vs. no), hypertension (yes vs. no), smoking (yes vs. no) in addition to 1,25(OH)_2_D levels < 41 pg/ml and BPA levels ≥ 110 ng/dl and expressed as odds ratio (OR) and corresponding 95% confidence intervals (95% CI). In additional logistic regression models, considering SCORE2/SCORE-2-OP of ≥ 20% or CV risk score of ≥ 3 as dependent variables separately, the association between independent variables 25(OH)D levels < 20 ng/ml, 1,25(OH)_2_D levels < 41 pg/ml and BPA levels ≥ 110 ng/dl were examined separately and together in specific models. Results from logistic regression were presented as odds ratio (OR) and relative 95% confidence intervals (CI). The effect modification between BPA and 25(OH)D or 1,25(OH)_2_D to explain the variability of CV risk score or SCORE-2/SCORE2-OP was investigated by linear and ordinal logistic regression, respectively, adjusting for age, gender and BMI. Receiver operating characteristic curve (ROC) analysis was applied to determine whether vitamin D [25(OH)D or 1,25(OH)_2_D] or BPA levels could be used to discriminate between individuals at very high CV risk as reflected by a CV risk score ≥ 3 or SCORE2/SCORE2-OP ≥ 20% and represented by area under the curve (AUC), sensitivity and specificity and relative 95% CI. A *p*-value of < 0.05 was considered statistically significant. Statistical analysis was performed using Instat Software (GraphPad, San Diego, CA, USA) or MedCalc Software (Broekstraat, Mariakerke, Belgium).

## Results

### Clinical and biochemical characteristics

Blood and urine samples from 299 elderly individuals (median age of 80 years; 35–99 years) were analysed and general demographic and laboratory variables are presented in Table 1. In this elderly population, 55.2% were female (*N* = 165). Patients were burdened with a high frequency of different comorbid diseases such as dyslipidaemia (48.2%), hypertension (47.5%), positive history of fall/fracture (29.4%) and 18.7% had obesity (BMI ≥ 30 kg/m^2^). Almost half of all patients were receiving some form of medication, mainly anti-hypertensive (52.2%), or anti-thrombotic (34.8%) drugs.

**Table 1:** Clinical and laboratory characteristics of individuals stratified by 25(OH)D levels

Characteristic	Total (*N* = 299)	25 (OH)D < 20 ng/ml (*N* = 180)	25 (OH)D ≥ 20 ng/ml (*N* = 119)	*p*-value
General				
Age, years (range)	80 (35–99)	81 (35–99)	72 (36–92)	** < 0.0001**
Female gender, n (%)	165 (55.2)	94 (52.2)	71 (59.7)	NS
BMI (kg/m^2^)	26.6 (13.4–41.2)	27.2 (13.4–41.2)	26.0 (14.9–38.7)	**0.0098**
Smoking status, *n* (%)				
Never	159 (53.2)	97(53.9)	62 (52.1)	NS
Previous	116 (38.8)	71 (39.4)	45 (37.8)	NS
Current	24 (8)	12 (6.7)	12 (10.1)	NS
Biochemical parameters				
1,25(OH)_2_D (pg/ml)	41.3 (12.4–136.0)	36.7 (12.4–87.5)	47 (15.9–136.0)	** < 0.0001**
25(OH)D (ng/ml)	17.2 (3.5–96.0)	13 (3.5–19.8)	27.9 (20.6–96.0)	** < 0.0001**
PTH (pmol/L)	5.0 (1.2–29.5)	5.5 (1.2–29.5)	4.2 (1.3–11.4)	** < 0.0001**
VDBP (mg/L)	680.4 (351–2597)	666.3 (351–2597)	708.1 (382–1267)	0.36
Creatinine (mg/dL)	0.78 (0.14–2.77)	0.62 (0.14–2.11)	0.94 (0.29–2.77)	0.48
BPA (ng/dL)	110 (27–2807)	179.5 (27–915)	51 (27–2807)	** < 0.0001**
Total calcium (mg/dL)	9.2 (7.9–10.6)	9.1 (7.9–10.6)	9.2 (8.3–10.3)	**0.0095**
Magnesium (mg/dl)	2.1 (1.2–2.8)	2.1 (1.6–2.4)	2.1 (1.5–2.8)	0.32
Total cholesterol (mg/dL)	212 (105–542)	211.5 (105–378)	212 (125–542)	0.53
LDL-cholesterol (mg/dL)	131 (34–455)	130 (34–257)	132 (61–455)	0.31
HDL-cholesterol (mg/dL)	56 (24–115)	56 (29–109)	57.5 (24–115)	0.07
Triglycerides (mg/dL)	98 (29–761)	104 (32–761)	94 (29–263)	**0.026**
Plasma glucose (mg/dL)	92 (57–218)	94 (70–171)	90 (57–218)	0.058
Comorbid diseases, *n* (%)				
Dyslipidemia	144 (48.2)	87 (48.3)	57 (47.9)	NS
Hypertension	142 (47.5)	93 (51.7)	49 (41.2)	0.08
Fall/fracture/hospitalisation	88 (29.4)	56 (31.1)	31 (26.1)	NS
Obesity	56 (18.7)	44 (24.4)	12 (10.1)	**0.002**
Osteoporosis	48 (16.1)	19 (10.6)	29 (24.4)	**0.003**
Cardiovascular disease	42 (14.0)	28 (15.6)	14 (11.8)	0.4
Type-2 diabetes mellitus	39 (13.0)	29 (16.1)	10 (8.4)	0.06
Asthma and/or bronchitis	18 (6.0)	14 (7.8)	4 (3.4)	0.14
Treatment, *n* (%)				
Antihypertensive	156 (52.2)	104 (57.8)	52 (43.7)	**0.025**
Antithrombotic	104 (34.8)	70 (38.9)	34 (28.6)	0.08
Psychotropic	82 (27.4)	52 (28.9)	30 (25.2)	NS
Lipid lowering	73 (24.4)	48 (26.7)	25 (21.0)	0.27
Cardiac	42 (14.0)	28 (15.6)	14 (11.8)	** < 0.0001**
Anti-diabetic	33 (11.0)	23 (12.8)	10 (8.4)	0.26
Vitamin D supplementation	28 (9.4)	3 (1.7)	25 (21.0)	** < 0.0001**
Anti-fracture	15 (5.0)	5 (2.8)	10 (8.4)	**0.05**

Statistically significant *p*-values (<0.05) are indicated in bold

Data are presented as number and % or mean and SD. *BMI* Body mass index; *BPA* Bisphenol A; *HDL* High-density lipoprotein; *LDL* Low-density lipoprotein; *PTH* Parathyroid hormone; *VDBP* Vitamin D binding protein

### Characteristics of individuals deficient (< 20 ng/ml) in 25(OH)D

Overall, 180 (60.2%) individuals had 25(OH)D levels < 20 ng/ml, a level that is considered deficient according to the Endocrine Society. Those with vitamin D deficiency tended to be older and have higher BMI, while gender and smoking status were similar to the non-vitamin D deficient group. As expected, lower levels of 1,25(OH)_2_D and 25(OH)D were associated with higher PTH levels. However, urinary BPA levels were significantly higher in the vitamin D deficient group (179.5 ng/dl vs. 51 ng/dl, *p* < 0.0001). Lower serum calcium and higher triglycerides were observed (104 mg/dl vs. 94 mg/dl; *p* = 0.026) in the vitamin D-deficient group compared to controls. Participants with vitamin D deficiency were more likely to be obese (i.e. BMI ≥ 30 kg/m^2^; 24.4% vs. 10.1%; *p* = 0.002) and less likely to have osteoporosis (10.6 vs. 24.4%; *p* = 0.002) and more likely to be taking anti-hypertensives (57.8% vs. 43.7%; *p* = 0.025), and cardiac drugs (15.6% vs. 11.8%; *p* < 0.0001). Of note, just 3 patients were not receiving any vitamin D supplementation (1.7% vs. 21%; *p* < 0.0001).

### Association between biochemical and clinical variables by univariate analysis

Statistically significant correlations were observed for a range of different variables (Supplementary Table 1). As previously observed [20], levels of BPA were highly (and negatively) correlated with 1,25(OH)_2_D (*r* = −0.67, *p* < 0.0001), 25(OH)D (*r* = −0.69, *p* < 0.0001) and PTH levels (*r* = −0.44, *p* < 0.0001). We also noted that BPA was positively correlated with triglyceride (*r* = 0.13, *p* = 0.03) and glucose levels (*r* = 0.21, *p* = 0.0003) and inversely correlated with serum calcium levels (*r* = −0.14, *p* = 0.03). Interestingly, levels of 1,25(OH)_2_D and 25(OH)D were negatively correlated with some of these variables to a similar extent.

### CV risk and correlation with vitamin D and BPA levels by univariate regression

**Fig. 1:**
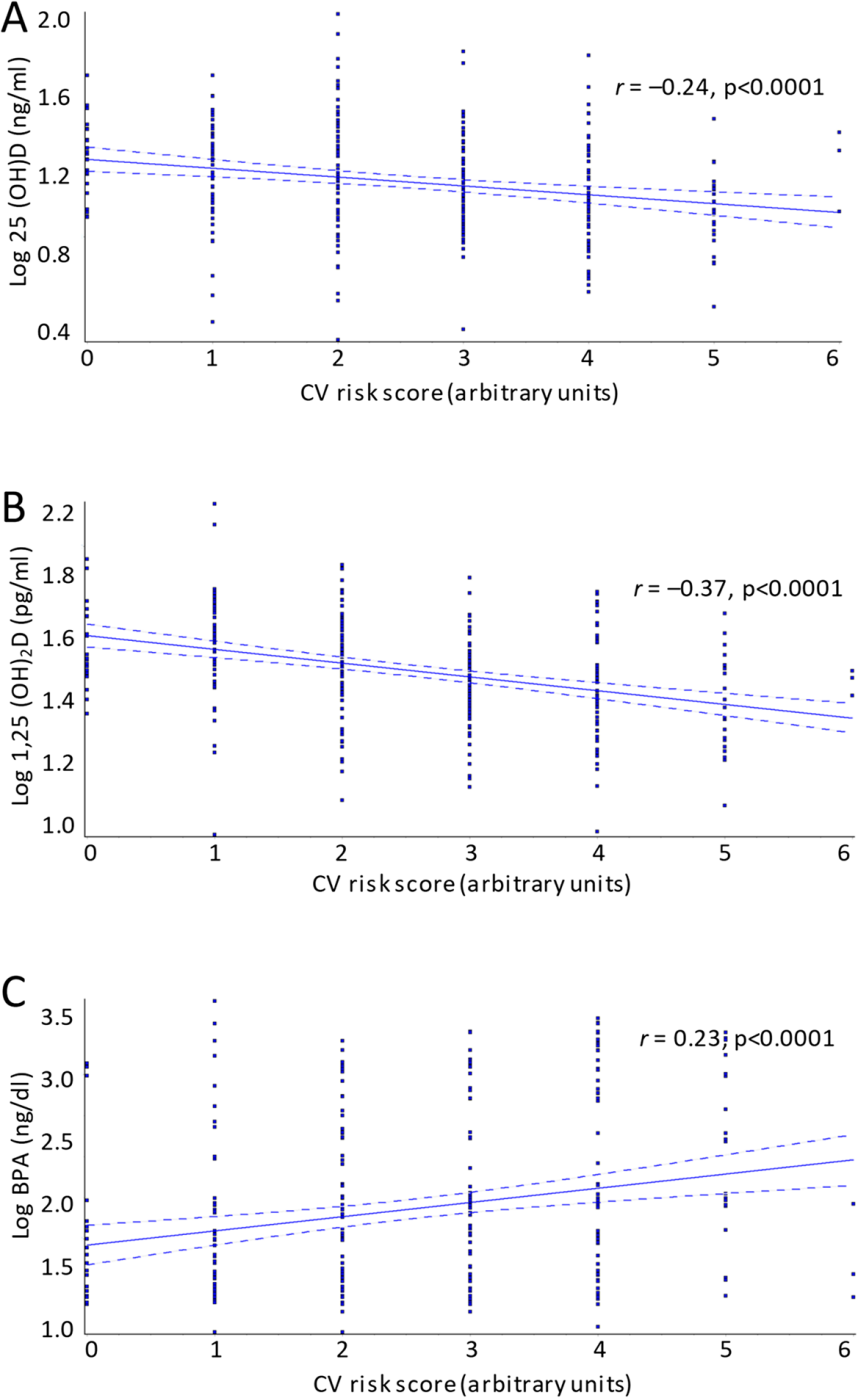
Correlation between CV risk score and serum vitamin D and BPA levels in 299 elderly individuals from the inChianti database. BPA = bisphenol A, CV = cardiovascular, r = regression coefficient

Using the arbitrary CV risk score, the mean CV risk score for the entire cohort was 2.6 ± 1.4 and levels of both the vitamin D metabolites 25(OH)D and 1,25(OH)_2_D were negatively correlated with CV risk score (r = −0.24, *p* < 0.0001 and r = −0.37; *p* < 0.0001 respectively) (Fig. 1A and B), whereas BPA levels were positively correlated with CV risk score (r = 0.23, *p* < 0.0001) (Fig. 1C). As expected, in individuals deficient in 25(OH)D, (< 20 ng/ml), CV risk score was significantly higher (2.8 ± 1.4) compared to individuals with 25(OH)D levels ≥ 20 ng/ml (2.2 ± 1.4, *p* < 0.0001).

Using the ESC-based algorithm, SCORE2/SCORE2-OP, the mean 10-year risk of a CV event for the entire cohort was 17.95 ± 12.5% (95% CI: 16.5–19.4%). Approximately one-third of patients (*N* = 104; 34.8%) had < 10% risk, 65.2% (*N* = 195) a ≥ 10% risk and just under half of the cohort (*N* = 142; 47.5%) a ≥ 20% risk of CV event in the next 10-years. Results from both scores for the entire cohort were positively correlated with each other (*r* = 0.52, *p* < 0.0001) (Supplementary Fig. 1).

**Fig. 2:**
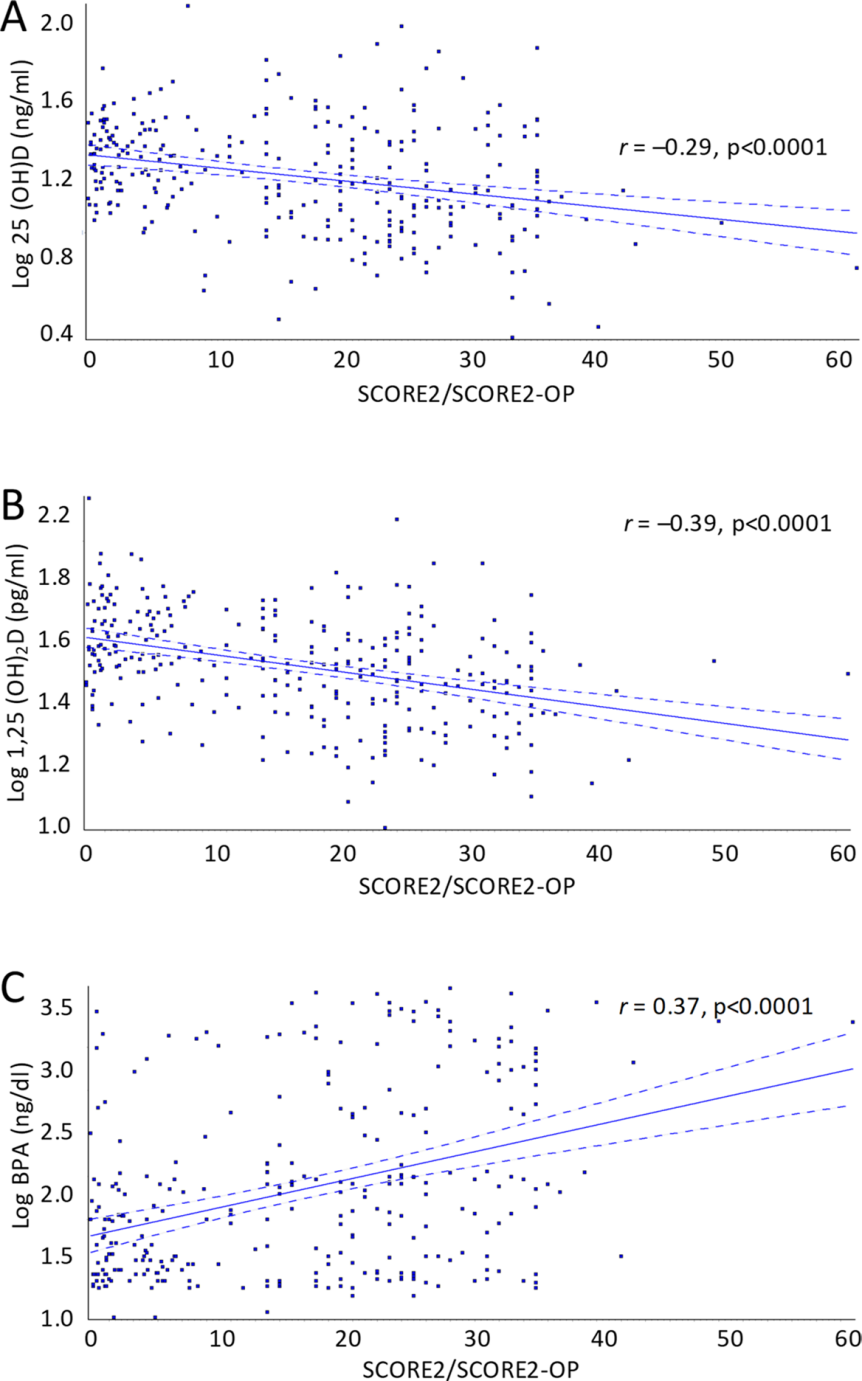
Correlation between SCORE2/SCORE2-OP and serum vitamin D and BPA levels in 299 elderly individuals from the inChianti database. BPA = bisphenol A, CV = cardiovascular, r = regression coefficient

Similar to results with CV risk score, levels of both 25(OH)D and 1,25(OH)_2_D were negatively correlated with SCORE2/SCORE2-OP (*r* = −0.29, *p* < 0.0001 and r = −0.39; *p* < 0.0001 respectively) (Fig. 2A and B) while BPA levels were positively correlated with SCORE2/SCORE2-OP (*r* = 0.37, *p* < 0.0001) (Fig. 2C). Likewise, in individuals deficient in 25(OH)D, (< 20 ng/ml), SCORE2/SCORE2-OP was significantly higher (20.5 ± 12.6%) compared to individuals with 25(OH)D levels ≥ 20 ng/ml (14.1 ± 11.4%, *p* < 0.0001).

**Fig. 3:**
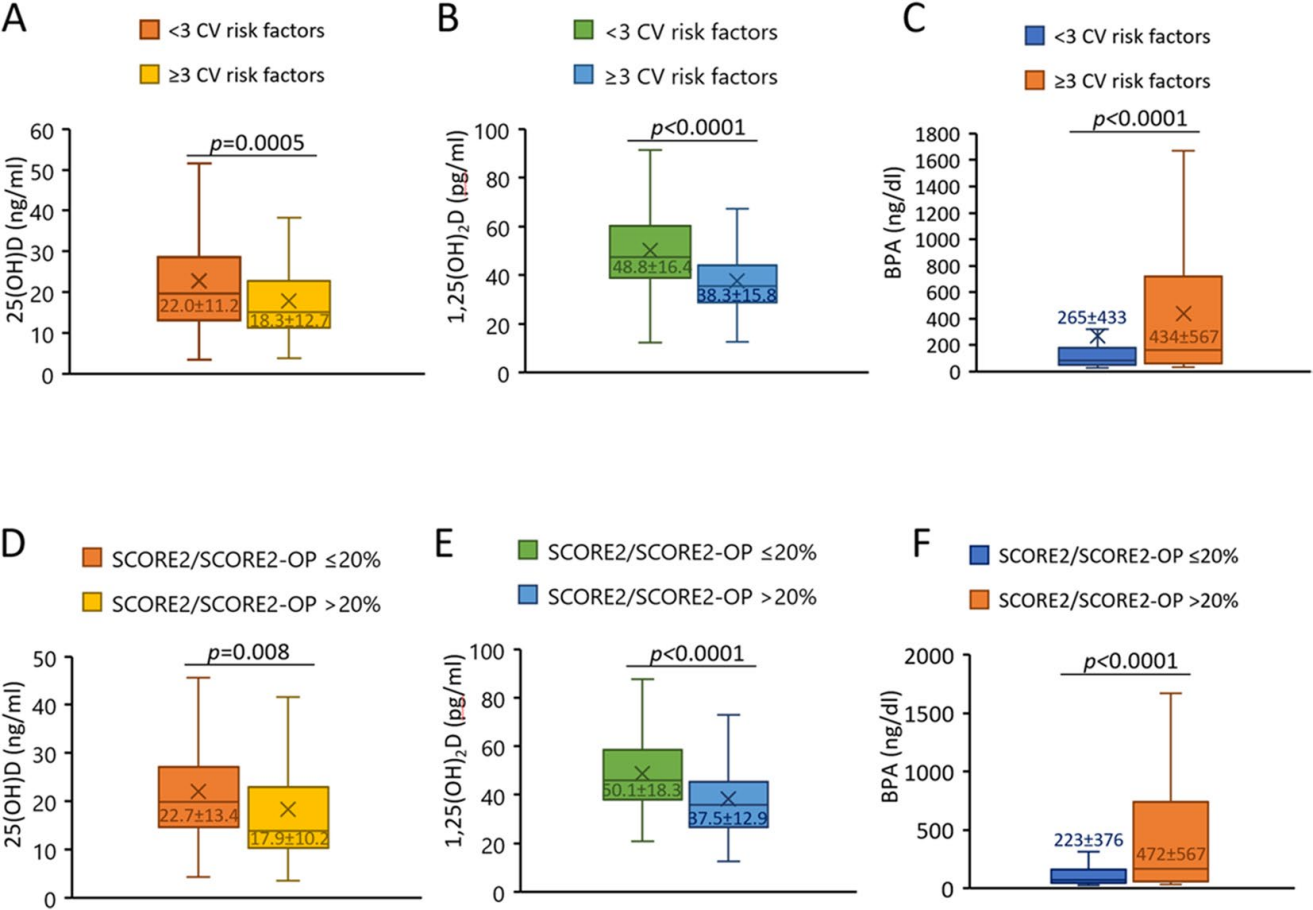
Comparison between levels of serum vitamin D and BPA in 299 elderly individuals from the inChianti database with very high CV risk reflected by a CV risk score ≥ 3 or SCORE2/SCORE2-OP ≥ 20%. BPA = bisphenol A, CV = cardiovascular, r = regression coefficient

Stratifying CV risk score and SCORE2/SCORE2-OP by median cut-off values (3 and 20% respectively), significant differences in the levels of vitamin D metabolites and BPA were observed (Fig. 3) whereby levels of both 25(OH)D and 1,25(OH)2D were significantly lower with higher risk scores and BPA levels showed the opposite, i.e. higher with increased CV risk (Fig. 3).

### Effect modification between vitamin D metabolites and BPA to explain CV risk

The mutual effect modification between BPA and 25(OH)D or 1,25(OH)_2_D to explain the variability of CV risk score and SCORE2/SCORE-2 was investigated by linear and ordinal logistic regression, respectively, adjusting for age, gender, and BMI. These analyses showed that no mutual effect modification exists between BPA and 25(OH)D or 1,25(OH)_2_D for explaining SCORE-2/ SCORE2-OP and CV risk score (all *p* > 0.7). This implies that the effect of BPA levels on SCORE-2/SCORE2-OP and CV-risk score is constant (i.e. it does not significantly change) across 1,25(OH)_2_D or 25(OH)D levels and that effect of 1,25(OH)_2_D or 25(OH)D levels on SCORE-2/ SCORE2-OP and CV-risk score is constant (i.e. it does not significantly change) across BPA values.

### Predictor variables associated with deficient levels of 25(OH)D (< 20 ng/ml)

Multivariate logistic regression analysis was next used to determine the direction and strength of the association between traditional CV risk factors as well as low 1,25(OH)_2_D and high BPA levels as predictor variables influencing 25(OH)D levels < 20 ng/ml. Including 25(OH)D < 20 ng/ml as dependent variable, from a range of predictor variables, BPA (OR: 20.9, 95% CI: 9.4–46.8, *p* < 0.0001) emerged as the strongest predictor associated with deficient levels of 25(OH)D levels (Table 2). Male gender (OR: 2.1, 95% CI: 1.1–3.8, *p* = 0.022) and BMI (OR: 2.8, 95% CI: 1.2–6.45, *p* = 0.016) were positively associated with 25(OH)D deficiency.

**Table 2:** Multivariate logistic regression analysis of variables associated with serum 25(OH)D levels < 20 ng/ml

Variable	OR (95% CI)	*p*-value
Model #1		
Age (≥ 75 years)	1.2 (0.6 − 2.4)	0.60
Gender (male)	2.1 (1.1–3.8)	**0.022**
BMI (≥ 30 kg/m^2^)	2.8 (1.2 − 6.45)	**0.016**
Diabetes (yes/no)	0.89 (0.34 − 2.3)	0.80
Dyslipidemia (yes/no)	0.91 (0.49 − 1.64)	0.74
Hypertension (yes/no)	1.51 (0.77 − 2.97)	0.23
Smoking (yes/no)	0.42 (0.14 − 1.31)	0.14
1,25(OH)_2_D (< 41 pg/ml)	1.04 (0.49 − 2.2)	0.92
BPA (≥ 110 ng/dL)	20.9 (9.4 − 46.8)	** < 0.0001**

Statistically significant *p*-values (<0.05) are indicated in bold

*BMI* Body mass index; *BPA* Bisphenol A; *CV* Cardiovascular; *PTH* Parathyroid hormone; *VDBP* Vitamin D binding protein

### Association of vitamin D and BPA with CV risk score ≥ 3 and SCORE2/SCORE2-OP ≥ 20%

Having stratified both CV risk score and SCORE2/SCORE2-OP by median values (i.e. 3 and 20% respectively) we next wanted to evaluate the association between vitamin D metabolites and BPA with very high CV risk. In separate models, deficient levels of 25(OH)D, 1,25(OH)_2_D levels of < 41 pg/ml and high BPA levels (≥ 110 ng/dl) were significantly associated with a CV risk score of 3 (Table 3). In a second model, including all three variables, both deficient levels of 25(OH)D and low 1,25(OH)_2_D levels remained statistically significant whereas the effect of BPA was lost. This analysis was repeated with SCORE2/SCORE2-OP with similar results observed. While all three variables (low vitamin D metabolites and high BPA) were associated with very high SCORE2/SCORE2-OP (i.e. ≥ 20%), only 1,25(OH)_2_D levels of < 41 pg/ml remained statistically significant when these three variables were considered together (Table 4).

**Table 3:** Multivariate linear regression analysis of variables associated with CV risk score ≥ 3

Variable	OR (95% CI)	*p*-value
Model #1		
25(OH)D (< 20 ng/ml)	2.4 (1.4 − 3.6)	**0.0008**
Model #2		
1,25(OH)_2_D (< 41 pg/ml)	3.89 (2.39 − 6.3)	** < 0.0001**
Model #3		
BPA (≥ 110 ng/dL)	1.87 (1.18 − 2.9)	**0.0079**
Model #4		
25(OH)D (< 20 ng/ml)	1.86 (1.02 − 3.39)	**0.044**
1,25(OH)_2_D (< 41 pg/ml)	4.16 (2.32 − 7.4)	** < 0.0001**
BPA (≥ 110 ng/dL)	0.6 (0.31 − 1.2)	0.56

Statistically significant *p*-values (<0.05) are indicated in bold

*BMI* Body mass index; *BPA* Bisphenol A; *CV* Cardiovascular; *PTH* Parathyroid hormone; *VDBP* Vitamin D binding protein

**Table 4:** Multivariate linear regression analysis of variables associated with SCORE2/SCORE2-OP ≥ 20%

Variable	OR (95% CI)	*p*-value
Model #1		
25(OH)D (< 20 ng/ml)	2.4 (1.5 − 3.9)	**0.0003**
Model #2		
1,25(OH)_2_D (< 41 pg/ml)	3.75 (2.3 − 6.1)	** < 0.0001**
Model #3		
BPA (≥ 110 ng/dL)	2.69 (1.68 − 4.3)	** < 0.0001**
Model #4		
25(OH)D (< 20 ng/ml)	1.51 (0.83 − 2.7)	0.18
1,25(OH)_2_D (< 41 pg/ml)	2.98 (1.7 − 5.2)	**0.0001**
BPA (≥ 110 ng/dL)	1.2 (0.64 − 2.3)	0.56

Statistically significant *p*-values (<0.05) are indicated in bold

*BMI* Body mass index; *BPA* Bisphenol A; *CV* Cardiovascular; *PTH* Parathyroid hormone; *VDBP* Vitamin D binding protein

### ROC analysis to predict cut-off concentrations of vitamin D and BPA to predict high CV risk

**Fig. 4:**
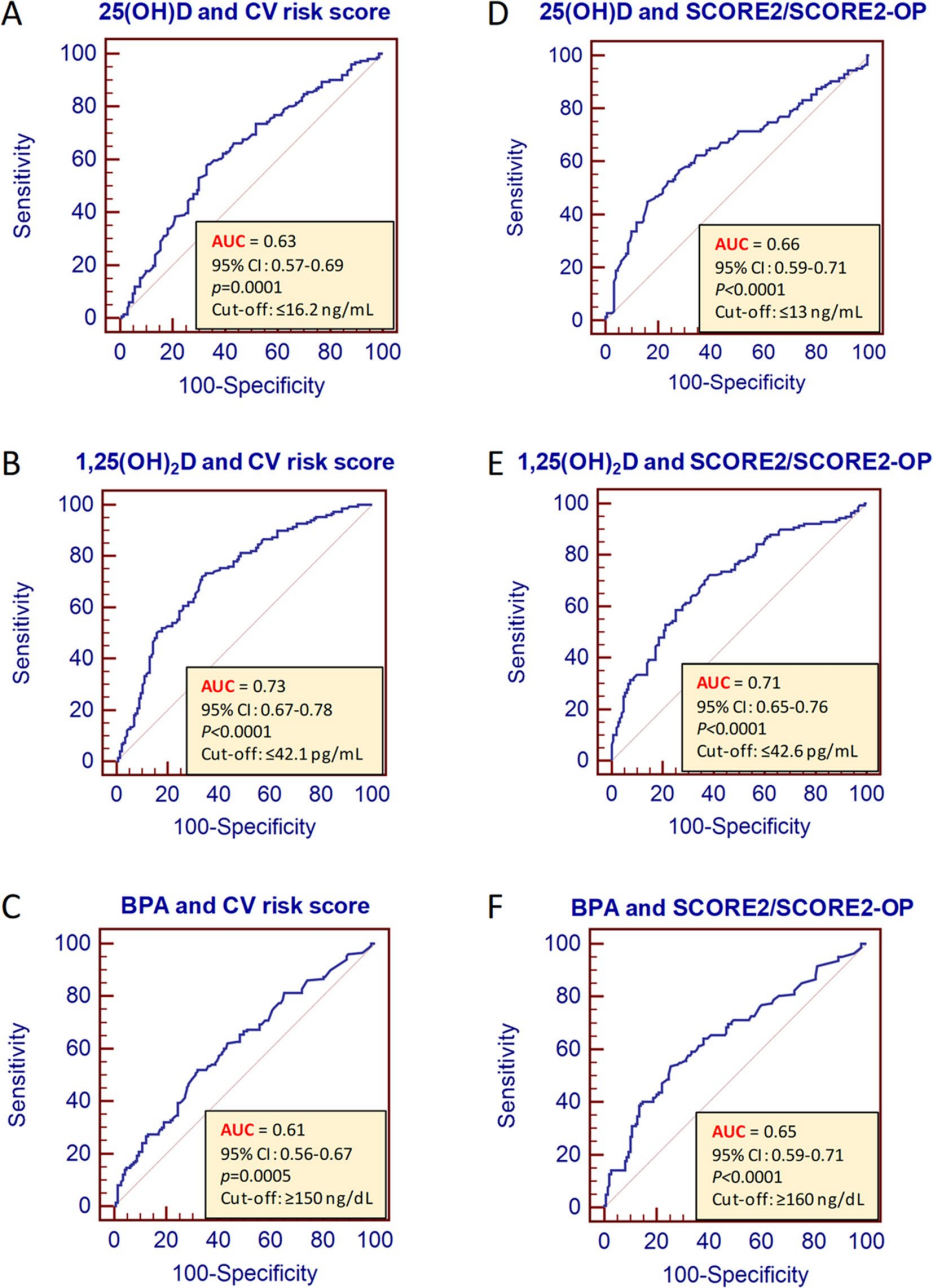
ROC analysis to assess the ability of vitamin D and BPA levels to discriminate between individuals at very high CV risk reflected by a CV risk score ≥ 3 or SCORE2/SCORE2-OP ≥ 20%. AUC = area under the curve, BPA = bisphenol A, CI = confidence interval, CV = cardiovascular

We used ROC analysis to assess the predictive power of vitamin D or BPA levels to discriminate individuals with low vs high CV risk (reflected by reflected by a CV risk score ≥ 3 or SCORE2/SCORE2-OP ≥ 20%) (Fig. 4). This analysis revealed that a 25(OH)D concentration of ≤ 16.2 ng/mL was the best cut-off to predict individuals with ≥ 3 risk factors for CV disease (AUC: 0.63, 95% CI: 0.57–0.69, *p* = 0.0001) (sensitivity = 57.6%, 95% CI: 49.3–65.6 and specificity of 67.1%, 95% CI: 58.8–74.8%) (Fig. 4A) while 1,25(OH)_2_D levels of < 42.1pg/ml was found to be the best cut-off to predict individuals with ≥ 3 risk factors for CV (AUC: 0.73, 95% CI: 0.67–0.78, *p* < 0.0001) (sensitivity = 72% (95% CI: 64.1–79% and specificity of 66.4%, 95% CI: 58.2–74%) (Fig. 4B). In addition, a BPA concentration of ≥ 150 ng/dl was found to be best cut-off to predict individuals with ≥ 3 risk factors for CV disease (AUC: 0.61, 95% CI: 0.56–0.67, *p* = 0.0005) (sensitivity = 52.0%, 95% CI: 43.7–60.2% and specificity of 68.0%, 95% CI: 59.8–75.5%) (Fig. 4C).

This analysis was also repeated to identify the best cut-off value to predict 10-year risk of a CV event according to SCORE2/SCORE2-OP. We identified that a 25(OH)D concentration of ≤ 13 ng/mL was the best cut-off to predict individuals with ≥ 20% 10-year CV risk according to SCORE2/SCORE2-OP algorithms (AUC: 0.66, 95% CI: 0.59–0.71, *p* < 0.0001) (sensitivity = 44.8%, 95% CI: 36.4–53.3% and specificity of 84.1%, 95% CI: 74.3–87.3%) (Fig. 4D) and for 1,25(OH)_2_D, < 42.6 pg/ml was identified as the best cut-off (AUC: 0.71, 95% CI: 0.65–0.76, *p* < 0.0001) (sensitivity = 72.1%, 95% CI: 63.9–79.4% and specificity of 61.6%, 95% CI: 53.3–69.4%) (Fig. 4E) while a BPA concentration of ≥ 160 ng/dl was the best cut-off (AUC: 0.65, 95% CI: 0.59–0.71, *p* < 0.0001) (sensitivity = 53.5%, 95% CI: 45.0–61.9% and specificity of 74.7%, 95% CI: 66.9–81.4%) (Fig. 4F).

## Discussion

The present follow-up analysis of our previous retrospective study [20] extends our understanding of the association between BPA and vitamin D and the potential impact on CV risk factors in an elderly population in Italy. While our previous analysis revealed a strong inverse relationship between urinary BPA concentrations and circulating serum vitamin D levels, confirming previous studies [21, 22, 33], the present analysis has gone further and explored a wide range of CV risk factors to determine whether alterations in levels of circulating vitamin D metabolites and exposure to BPA could impact upon CV risk.

It is recognised that vitamin D exerts effects beyond bone and mineral metabolism [1]. In some studies low serum concentrations of 25(OH)D were found to be associated with a higher incidence of CVD [34, 35], in addition to the known associations of risk factors for CVD, such as dyslipidaemia, smoking, diabetes mellitus, obesity, and hypertension [36, 37]. Although there is accumulating evidence that circulating levels of vitamin D is associated with many health outcomes, however, data from many studies suggests that vitamin D supplementation is ineffective in the prevention of most of these outcomes. This is a paradox that still remains to be clarified. Indeed, it is possible that vitamin D could be a biomarker of some other hidden condition that is causal in CV disease and may also affect vitamin D as a biomarker.

Indeed, in large meta-analyses of observational studies a negative correlation between serum 25(OH)D levels and risk of CVD have been observed [38–41]. However, interpretation of these findings may be limited by reverse causality or uncontrolled confounding. In four of these Mendelian randomization studies (which were designed to avoid these biases), two studies reported an inverse association between genetically predicted 25(OH)D levels up to 50 nmol/l (20 ng/ml) and CVD [42], including mortality [13]. In the latter study by Sutherland and colleagues [13], the relationship between 25(OH)D and mortality was investigated in a large prospective study performed in the UK that included 307,601 individuals. The main finding that emerged from this analysis revealed a non-linear relationship between 25(OH)D levels (adjusted for confounders) and the odds of all-cause mortality in the genetic analysis were estimated to increase by 25% (OR: 1.25, 95% CI: 1.16–1.35) for participants with 25(OH)D levels of 25 nmol/L vs. 50 nmol/L. Of note, the risk of mortality steeply increased with decreasing 25(OH)D levels below 50 nmol/L (20 ng/ml) and similar results were also observed for other endpoints such as cancer, CV and respiratory-related mortality [13]. However, other studies found no association, but did not allow for nonlinear effects [43, 44]. It is important to note that recent evidence has also questioned the validity of the statistical methods used in Mendelian randomisation analysis and consequently the results obtained from some of these studies [45].

A lack of effect of vitamin D supplementation on CV events has also been observed in other types of studies. A meta-analysis of randomised controlled trials concluded that vitamin D supplementation did not prevent cardiovascular events [46]. However, 45% of the 83,291 participants included in the meta-analysis used a low dose of vitamin D, and had relatively low compliance [47]. CVD was the primary outcome of the Vitamin D Assessment (ViDA) study [48] and the Vitamin D and Omega 3 trial (VITAL) [49]. Despite different outcome definitions, both RCTs concluded that vitamin D supplementation had no effect on CVD [48, 49], but VITAL excluded people with a history of CVD and the ViDA study had relatively few events.

In the D-Health Trial [50] it was observed that vitamin D supplementation did not reduce all-cause mortality or mortality due to CVD [50], however, a slight (but non-significant) benefit afforded by vitamin D supplementation was observed in a recent follow-up analysis on the incidence of major CV events [51].

While we have previously discussed in detail the negative correlation between BPA and vitamin D levels [20], an association that has also been previously described elsewhere [21, 22, 33], the present analysis of 299 samples revealed a strong and highly significant inverse correlation between serum 1,25(OH)_2_D and 25(OH)D with CV risk and a positive correlation between urinary BPA and an arbitrary CV risk score. Using variables that are recognised to be associated with CV risk and included in other validated measures such as the Framingham Score [27, 28], and also used in other settings [26, 52], we were able to quantify the relative burden of CV risk in each patient and then determine if levels of a range of biochemical measures influenced this CV risk score. More importantly, these same results observed using an arbitrary CV risk score were also observed using SCORE2/SCORE2-OP, a validated measure of 10-year CV risk [29, 30]. The observation that lower levels of vitamin D were associated with higher CV risk confirms evidence using specific CV endpoints as discussed previously from trials and meta-analysis studies, but to our knowledge no study has yet documented this association.

Besides the inverse association observed between circulating vitamin D levels and CV risk, we also observed a strong positive correlation between BPA levels and CV risk score and SCORE2/SCORE2-OP. As the most widely consumed EDC, BPA has previously been linked to reproductive dysfunction, diabetes and obesity [16, 17]. However, few studies have specifically investigated the association between BPA and CVD.

Epidemiological studies based on NHANES 2003–2004 and 2005–2006 data revealed an association between BPA and CVD [53, 54]. Furthermore, a study based in the UK also found that BPA increases the CVD risk [55]. However, two studies, one using NHANES 2003–2010 data and another from Spain, did not observe an association between BPA and CVD [56, 57].

From the NHANES database (2003–2016) based on 11,857 adults, Moon et al. evaluated the association between BPA and CVD [58]. After adjusting for age, sex, race/ethnicity, BMI, cigarette smoking, diabetes status, hypertension, and dyslipidaemia, the OR between logarithmic transformed BPA and CVD was 1.13 (95% CI: 1.02–1.24; *p* = 0.01).

It is important to highlight that vitamin D individuals accounted for 60% of the present cohort and in this population only 3 patients were currently receiving vitamin D supplementation and in the remaining group (levels above the threshold of 20 ng/ml), only 21% (*N* = 25) of individuals were receiving vitamin D supplementation. This low number can be likely explained by the fact that many patients who had deficient or severely deficient levels of vitamin D would have received vitamin D supplementation thereby correcting levels to the normal range. Regardless, considering the high burden of comorbid diseases and advanced age of these individuals it is of concern that 60% of patients were vitamin D deficient (also considering that almost 30% had a fracture or fall within the past year) and were not receiving vitamin D or anti-fracture supplementation.

Vitamin D was also negatively correlated (by univariate analysis) with BMI and obesity was associated with deficient levels of 25(OH)D by multivariate analysis, confirming previous studies [59, 60]. It is well documented that there is a high prevalence of vitamin D deficiency in obese subjects and this may be explained by the volumetric dilution into the greater volumes of fat, serum, liver, and muscle present in obese people [61]. However, other mechanisms cannot completely be excluded. In a meta-analysis study by Zimmerman and colleagues [62], the importance of body weight for the dose–response relationship with circulating 25(OH)D has shown that 34.5% of variation in circulating 25(OH)D was explained by body weight. In addition to obesity, male gender was also associated with deficient vitamin D levels, a finding that has also been observed elsewhere [63].

In this study, elderly individuals may be representative of the general population, particularly for this age category and a high proportion (60%) were deficient in 25(OH)D (i.e. < 20 ng/ml), and as such are at increased risk of bone-related diseases such as fracture [1] as well as being burdened with comorbid diseases. The interrelationship between long term-exposure of BPA and circulating vitamin D levels and potential impact upon increased CV risk needs to be further characterised. In the short-term, strategies to avoid and or limit BPA exposure as well as monitor for vitamin D deficiency in elderly individuals should be implemented.

### Study limitations

There are some limitations of the present study that need to be highlighted. First, this analysis was cross-sectional and no follow-up data were considered. Therefore, associations observed were strictly hypothesis generating and no causality can be claimed. Although the population included in this analysis were homogenous in terms of clinical characteristics, we were still able to detect important and significant associations among variables examined. No hard end-points such as fracture or mortality were assessed. Despite this, by using a cumulative risk score of recognised CV risk factors and a validated measure of CV risk (SCORE2/SCORE2-OP), we were able to show how increased BPA levels can be linked to low vitamin D and CV risk.

## Conclusion

In this cross-sectional analysis of an elderly population, levels of the EDC BPA were found to negatively impact upon levels of serum vitamin D as well as being correlated with CV risk. Future follow-up analysis examining hard endpoints such as BMD, fracture and mortality will improve our understanding of the long-term impact of BPA exposure in the elderly.

## Supplementary Information

Below is the link to the electronic supplementary material.

Supplementary file1 (PPTX 155 KB)

Supplementary file2 (DOCX 16.7 KB)

## Data Availability

Data can be provided by the corresponding author upon request.

## References

[1] Bouillon R, Marcocci C, Carmeliet G, et al. Skeletal and extraskeletal actions of vitamin D: current evidence and outstanding questions. Endocr Rev. 2019;40:1109–51. 10.1210/er.2018-00126. 30321335 10.1210/er.2018-00126 PMC6626501

[2] Holick MF, Binkley NC, Bischoff-Ferrari HA, et al. Evaluation, treatment, and prevention of vitamin D deficiency: an Endocrine Society clinical practice guideline. J Clin Endocrinol Metab. 2011;96:1911–30. 10.1210/jc.2011-0385. 21646368 10.1210/jc.2011-0385

[3] Minisola S, Colangelo L, Pepe J, et al. Osteomalacia and vitamin D status: a clinical update 2020. JBMR Plus. 2021;5: e10447. 10.1002/jbm4.10447. 33553992 10.1002/jbm4.10447 PMC7839817

[4] Giannini S, Giusti A, Minisola S, et al. The immunologic profile of vitamin D and its role in different immune-mediated diseases: an expert opinion. Nutrients. 2022;14:473. 10.3390/nu14030473. 35276834 10.3390/nu14030473 PMC8838062

[5] Palacios C, Gonzalez L. Is vitamin D deficiency a major global public health problem? J Steroid Biochem Mol Biol. 2014;144 Pt A:138–45. 10.1016/j.jsbmb.2013.11.003. 24239505 10.1016/j.jsbmb.2013.11.003 PMC4018438

[6] Amrein K, Scherkl M, Hoffmann M, et al. Vitamin D deficiency 2.0: an update on the current status worldwide. Eur J Clin Nutr. 2020;74:1498–513. 10.1038/s41430-020-0558-y. 31959942 10.1038/s41430-020-0558-y PMC7091696

[7] Hill TR, Aspray TJ. The role of vitamin D in maintaining bone health in older people. Ther Adv Musculoskelet Dis. 2017;9:89–95. 10.1177/1759720X17692502. 28382112 10.1177/1759720X17692502 PMC5367643

[8] Mithal A, Wahl DA, Bonjour J-P, et al. Global vitamin D status and determinants of hypovitaminosis D. Osteoporos Int J Establ Result Coop Eur Found Osteoporos Natl Osteoporos Found USA. 2009;20:1807–20. 10.1007/s00198-009-0954-6. 10.1007/s00198-009-0954-6 19543765

[9] Allain TJ, Dhesi J. Hypovitaminosis D in older adults. Gerontology. 2003;49:273–8. 10.1159/000071707. 12920346 10.1159/000071707

[10] Salamone LM, Dallal GE, Zantos D, et al. Contributions of vitamin D intake and seasonal sunlight exposure to plasma 25-hydroxyvitamin D concentration in elderly women. Am J Clin Nutr. 1994;59:80–6. 10.1093/ajcn/59.1.80. 8279408 10.1093/ajcn/59.1.80

[11] Giustina A, Bouillon R, Dawson-Hughes B, et al. Vitamin D in the older population: a consensus statement. Endocrine. 2023;79:31–44. 10.1007/s12020-022-03208-3. 36287374 10.1007/s12020-022-03208-3 PMC9607753

[12] Autier P, Boniol M, Pizot C, Mullie P. Vitamin D status and ill health: a systematic review. Lancet Diabetes Endocrinol. 2014;2:76–89. 10.1016/S2213-8587(13)70165-7. 24622671 10.1016/S2213-8587(13)70165-7

[13] Sutherland JP, Zhou A, Hyppönen E. Vitamin D deficiency increases mortality risk in the UK biobank : a nonlinear mendelian randomization study. Ann Intern Med. 2022;175:1552–9. 10.7326/M21-3324. 36279545 10.7326/M21-3324

[14] Bisphenol A - an overview | ScienceDirect Topics. https://www.sciencedirect.com/topics/chemistry/bisphenol-a. Accessed 27 Feb 2021

[15] Update on bisphenol a for use in food contact applications U.S. Food and Drug Administration January 2010

[16] Seachrist DD, Bonk KW, Ho S-M, et al. A review of the carcinogenic potential of bisphenol A. Reprod Toxicol Elmsford N. 2016;59:167–82. 10.1016/j.reprotox.2015.09.006. 10.1016/j.reprotox.2015.09.006 PMC4783235 26493093

[17] Gao X, Wang H-S. Impact of bisphenol A on the cardiovascular system — epidemiological and experimental evidence and molecular mechanisms. Int J Environ Res Public Health. 2014;11:8399–413. 10.3390/ijerph110808399. 25153468 10.3390/ijerph110808399 PMC4143868

[18] Fonseca MI, Lorigo M, Cairrao E. Endocrine-disrupting effects of bisphenol A on the cardiovascular system: a review. J Xenobiotics. 2022;12:181–213. 10.3390/jox12030015. 10.3390/jox12030015 PMC9326625 35893265

[19] Chen M, Yang Y, Baral K, et al. Relationship between bisphenol A and the cardiovascular disease metabolic risk factors in American adults: a population-based study. Chemosphere. 2023;324:138289. 10.1016/j.chemosphere.2023.138289. 36870620 10.1016/j.chemosphere.2023.138289

[20] Brandi ML, Bandinelli S, Iantomasi T, et al. Association between vitamin D and bisphenol A levels in an elderly Italian population: results from the InCHIANTI study. Endocr Connect. 2022;11:e210571. 10.1530/EC-21-0571. 35148277 10.1530/EC-21-0571 PMC8942328

[21] Johns LE, Ferguson KK, Meeker JD. Relationships between urinary phthalate metabolite and bisphenol A concentrations and vitamin D levels in U.S. adults: National Health and Nutrition Examination Survey (NHANES), 2005–2010. J Clin Endocrinol Metab. 2016;101:4062–9. 10.1210/jc.2016-2134. 27648964 10.1210/jc.2016-2134 PMC5095248

[22] Johns LE, Ferguson KK, Cantonwine DE, et al. Urinary BPA and phthalate metabolite concentrations and plasma vitamin D levels in pregnant women: a repeated measures analysis. Environ Health Perspect. 2017;125:087026. 10.1289/EHP1178. 28934718 10.1289/EHP1178 PMC5783673

[23] Ferrucci L, Bandinelli S, Benvenuti E, et al. Subsystems contributing to the decline in ability to walk: bridging the gap between epidemiology and geriatric practice in the InCHIANTI study. J Am Geriatr Soc. 2000;48:1618–25. 10.1111/j.1532-5415.2000.tb03873.x. 11129752 10.1111/j.1532-5415.2000.tb03873.x

[24] Ministero dell salute (2002) Italian legislative decree no. 6 del 2002 for the conduct of observational studies. In: Minist. Della Salute. https://www.aosp.bo.it/files/2002-09-02_0.pdf

[25] NHANES 2003–2004: environmental phenols data documentation, codebook, and frequencies. https://wwwn.cdc.gov/Nchs/Nhanes/2003-2004/L24EPH_C.htm. Accessed 22 Feb 2021

[26] Egan CG, Lavery R, Caporali F, et al. Generalised reduction of putative endothelial progenitors and CXCR4-positive peripheral blood cells in type 2 diabetes. Diabetologia. 2008;51:1296–305. 10.1007/s00125-008-0939-6. 18286257 10.1007/s00125-008-0939-6

[27] Grundy SM, Pasternak R, Greenland P, et al. Assessment of cardiovascular risk by use of multiple-risk-factor assessment equations: a statement for healthcare professionals from the American Heart Association and the American College of Cardiology. Circulation. 1999;100:1481–92. 10.1161/01.cir.100.13.1481. 10500053 10.1161/01.cir.100.13.1481

[28] Grundy SM, Cleeman JI, Merz CNB, et al. Implications of recent clinical trials for the National Cholesterol Education Program Adult Treatment Panel III guidelines. Circulation. 2004;110:227–39. 10.1161/01.CIR.0000133317.49796.0E. 15249516 10.1161/01.CIR.0000133317.49796.0E

[29] SCORE2 working group and ESC Cardiovascular risk collaboration. SCORE2 risk prediction algorithms: new models to estimate 10-year risk of cardiovascular disease in Europe. Eur Heart J. 2021;42:2439–54. 10.1093/eurheartj/ehab309. 34120177 10.1093/eurheartj/ehab309 PMC8248998

[30] SCORE2-OP working group and ESC Cardiovascular risk collaboration. SCORE2-OP risk prediction algorithms: estimating incident cardiovascular event risk in older persons in four geographical risk regions. Eur Heart J. 2021;42:2455–67. 10.1093/eurheartj/ehab312. 34120185 10.1093/eurheartj/ehab312 PMC8248997

[31] Mancia G, Kreutz R, Brunström M, et al. 2023 ESH Guidelines for the management of arterial hypertension The Task Force for the management of arterial hypertension of the European Society of Hypertension: Endorsed by the International Society of Hypertension (ISH) and the European Renal Association (ERA). J Hypertens. 2023;41:1874–2071. 10.1097/HJH.0000000000003480. 37345492 10.1097/HJH.0000000000003480

[32] Unger T, Borghi C, Charchar F, et al. 2020 international society of hypertension global hypertension practice guidelines. Hypertension. 2020;75:1334–57. 10.1161/HYPERTENSIONAHA.120.15026. 32370572 10.1161/HYPERTENSIONAHA.120.15026

[33] Erden ES, Genc S, Motor S, et al. Investigation of serum bisphenol A, vitamin D, and parathyroid hormone levels in patients with obstructive sleep apnea syndrome. Endocrine. 2014;45:311–8. 10.1007/s12020-013-0022-z. 23904340 10.1007/s12020-013-0022-z

[34] Demer LL, Hsu JJ, Tintut Y. Steroid hormone vitamin D: implications for cardiovascular disease. Circ Res. 2018;122:1576–85. 10.1161/CIRCRESAHA.118.311585. 29798901 10.1161/CIRCRESAHA.118.311585 PMC6122607

[35] Juonala M, Voipio A, Pahkala K, et al. Childhood 25-OH vitamin D levels and carotid intima-media thickness in adulthood: the cardiovascular risk in young Finns study. J Clin Endocrinol Metab. 2015;100:1469–76. 10.1210/jc.2014-3944. 25668290 10.1210/jc.2014-3944

[36] Abbasi F, Feldman D, Caulfield MP, et al. Relationship among 25-hydroxyvitamin D concentrations, insulin action, and cardiovascular disease risk in patients with essential hypertension. Am J Hypertens. 2015;28:266–72. 10.1093/ajh/hpu136. 25138785 10.1093/ajh/hpu136 PMC4357801

[37] OliaiAraghi S, van Dijk SC, Ham AC, et al. BMI and body fat mass is inversely associated with vitamin D levels in older individuals. J Nutr Health Aging. 2015;19:980–5. 10.1007/s12603-015-0657-y.26624208 10.1007/s12603-015-0657-y

[38] Zhang R, Li B, Gao X, et al. Serum 25-hydroxyvitamin D and the risk of cardiovascular disease: dose-response meta-analysis of prospective studies. Am J Clin Nutr. 2017;105:810–9. 10.3945/ajcn.116.140392. 28251933 10.3945/ajcn.116.140392

[39] Gholami F, Moradi G, Zareei B, et al. The association between circulating 25-hydroxyvitamin D and cardiovascular diseases: a meta-analysis of prospective cohort studies. BMC Cardiovasc Disord. 2019;19:248. 10.1186/s12872-019-1236-7. 31699030 10.1186/s12872-019-1236-7 PMC6836514

[40] Wang L, Song Y, Manson JE, et al. Circulating 25-hydroxy-vitamin D and risk of cardiovascular disease: a meta-analysis of prospective studies. Circ Cardiovasc Qual Outcomes. 2012;5:819–29. 10.1161/CIRCOUTCOMES.112.967604. 23149428 10.1161/CIRCOUTCOMES.112.967604 PMC3510675

[41] Elamin MB, Abu Elnour NO, Elamin KB, et al. Vitamin D and cardiovascular outcomes: a systematic review and meta-analysis. J Clin Endocrinol Metab. 2011;96:1931–42. 10.1210/jc.2011-0398. 21677037 10.1210/jc.2011-0398

[42] Zhou A, Selvanayagam JB, Hyppönen E. Non-linear Mendelian randomization analyses support a role for vitamin D deficiency in cardiovascular disease risk. Eur Heart J. 2022;43:1731–9. 10.1093/eurheartj/ehab809. 34891159 10.1093/eurheartj/ehab809

[43] Meng X, Li X, Timofeeva MN, et al. Phenome-wide Mendelian-randomization study of genetically determined vitamin D on multiple health outcomes using the UK Biobank study. Int J Epidemiol. 2019;48:1425–34. 10.1093/ije/dyz182. 31518429 10.1093/ije/dyz182 PMC6857754

[44] Huang T, Afzal S, Yu C, et al. Vitamin D and cause-specific vascular disease and mortality: a Mendelian randomisation study involving 99,012 Chinese and 106,911 European adults. BMC Med. 2019;17:160. 10.1186/s12916-019-1401-y. 31466528 10.1186/s12916-019-1401-y PMC6716818

[45] Endocrinology TE of TLD&. Retraction and republication—Estimating dose-response relationships for vitamin D with coronary heart disease, stroke, and all-cause mortality: observational and Mendelian randomisation analyses. Lancet Diabetes Endocrinol. 2024;12:8. 10.1016/S2213-8587(23)00364-9. 38048795 10.1016/S2213-8587(23)00364-9 PMC10834373

[46] Barbarawi M, Kheiri B, Zayed Y, et al. Vitamin D supplementation and cardiovascular disease risks in more than 83 000 individuals in 21 randomized clinical trials. JAMA Cardiol. 2019;4:765–75. 10.1001/jamacardio.2019.1870. 31215980 10.1001/jamacardio.2019.1870 PMC6584896

[47] Jackson RD, LaCroix AZ, Gass M, et al. Calcium plus vitamin D supplementation and the risk of fractures. N Engl J Med. 2006;354:669–83. 10.1056/NEJMoa055218. 16481635 10.1056/NEJMoa055218

[48] Scragg R, Stewart AW, Waayer D, et al. Effect of monthly high-dose vitamin D supplementation on cardiovascular disease in the vitamin D assessment study : a randomized clinical trial. JAMA Cardiol. 2017;2:608–16. 10.1001/jamacardio.2017.0175. 28384800 10.1001/jamacardio.2017.0175 PMC5815022

[49] Manson JE, Cook NR, Lee I-M, et al. Vitamin D supplements and prevention of cancer and cardiovascular disease. N Engl J Med. 2019;380:33–44. 10.1056/NEJMoa1809944. 30415629 10.1056/NEJMoa1809944 PMC6425757

[50] Neale RE, Baxter C, Romero BD, et al. The D-Health Trial: a randomised controlled trial of the effect of vitamin D on mortality. Lancet Diabetes Endocrinol. 2022;10:120–8. 10.1016/S2213-8587(21)00345-4. 35026158 10.1016/S2213-8587(21)00345-4

[51] Thompson B, Waterhouse M, English DR, et al. Vitamin D supplementation and major cardiovascular events: D-Health randomised controlled trial. BMJ. 2023;381:e075230. 10.1136/bmj-2023-075230. 37380191 10.1136/bmj-2023-075230 PMC10302209

[52] Egan CG, Caporali F, Capecchi PL, et al. Levels of circulating CXCR4-positive cells are decreased and negatively correlated with risk factors in cardiac transplant recipients. Heart Vessels. 2011;26:258–66. 10.1007/s00380-010-0053-9. 21052687 10.1007/s00380-010-0053-9

[53] Lang IA, Galloway TS, Scarlett A, et al. Association of urinary bisphenol A concentration with medical disorders and laboratory abnormalities in adults. JAMA. 2008;300:1303–10. 10.1001/jama.300.11.1303. 18799442 10.1001/jama.300.11.1303

[54] Melzer D, Rice NE, Lewis C, et al. Association of urinary bisphenol a concentration with heart disease: evidence from NHANES 2003/06. PLoS ONE. 2010;5:e8673. 10.1371/journal.pone.0008673. 20084273 10.1371/journal.pone.0008673 PMC2800195

[55] Melzer D, Osborne NJ, Henley WE, et al. Urinary bisphenol A concentration and risk of future coronary artery disease in apparently healthy men and women. Circulation. 2012;125:1482–90. 10.1161/CIRCULATIONAHA.111.069153. 22354940 10.1161/CIRCULATIONAHA.111.069153

[56] LaKind JS, Goodman M, Naiman DQ. Use of NHANES data to link chemical exposures to chronic diseases: a cautionary tale. PLoS ONE. 2012;7:e51086. 10.1371/journal.pone.0051086. 23227235 10.1371/journal.pone.0051086 PMC3515548

[57] Salamanca-Fernández E, Rodríguez-Barranco M, Petrova D, et al. Bisphenol A exposure and risk of ischemic heart disease in the Spanish European Prospective Investigation into cancer and nutrition study. Chemosphere. 2020;261:127697. 10.1016/j.chemosphere.2020.127697. 32731019 10.1016/j.chemosphere.2020.127697

[58] Moon S, Yu SH, Lee CB, et al. Effects of bisphenol A on cardiovascular disease: an epidemiological study using National Health and Nutrition Examination Survey 2003–2016 and meta-analysis. Sci Total Environ. 2021;763:142941. 10.1016/j.scitotenv.2020.142941. 33158523 10.1016/j.scitotenv.2020.142941

[59] Guagnano MT, D’Ardes D, Di Giovanni P, et al. Gender, obesity, fat distribution and 25-Hydroxyvitamin D. Med Kaunas Lith. 2023;59:1123. 10.3390/medicina59061123. 10.3390/medicina59061123 PMC10304511 37374327

[60] Arunabh S, Pollack S, Yeh J, Aloia JF. Body fat content and 25-hydroxyvitamin D levels in healthy women. J Clin Endocrinol Metab. 2003;88:157–61. 10.1210/jc.2002-020978. 12519845 10.1210/jc.2002-020978

[61] Vranić L, Mikolašević I, Milić S. Vitamin D deficiency: consequence or cause of obesity? Medicina (Mex). 2019;55:541. 10.3390/medicina55090541. 10.3390/medicina55090541 PMC6780345 31466220

[62] Zimmerman D, Sood MM, Rigatto C, et al. Systematic review and meta-analysis of incidence, prevalence and outcomes of atrial fibrillation in patients on dialysis. Nephrol Dial Transplant Off Publ Eur Dial Transpl Assoc - Eur Ren Assoc. 2012;27:3816–22. 10.1093/ndt/gfs416. 10.1093/ndt/gfs416 23114904

[63] Sanghera DK, Sapkota BR, Aston CE, Blackett PR. Vitamin D status, gender differences and cardiometabolic health disparities. Ann Nutr Metab. 2017;70:79–87. 10.1159/000458765. 28315864 10.1159/000458765 PMC5480371

