# Supplementary material for: Association of vitamin D and bisphenol A levels with cardiovascular risk in an elderly Italian population: results from the InCHIANTI study
Source: GeroScience. 2024 Jun 5;46(6):6141–56. doi: 10.1007/s11357-024-01193-1 (PMC11494005; doi:10.1007/s11357-024-01193-1)
Supplement: Supplementary file 1 — Supplementary file1 (PPTX 155 KB) [file 11357_2024_1193_MOESM1_ESM.pptx]

## Slide 1
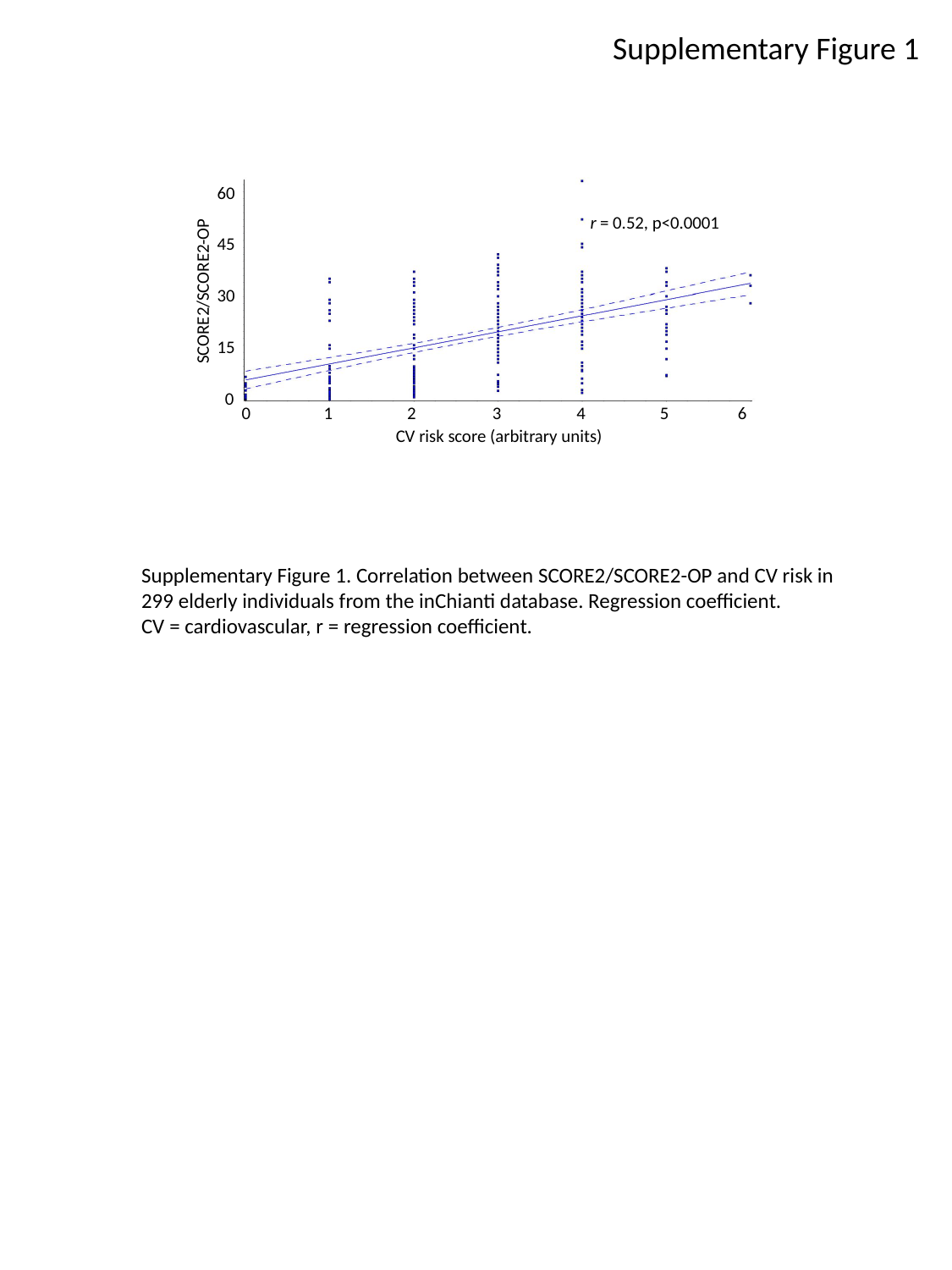

Supplementary Figure 1
60
r = 0.52, p<0.0001
45
SCORE2/SCORE2-OP
30
15
0
0
1
2
3
4
5
6
CV risk score (arbitrary units)
Supplementary Figure 1. Correlation between SCORE2/SCORE2-OP and CV risk in
299 elderly individuals from the inChianti database. Regression coefficient.
CV = cardiovascular, r = regression coefficient.
