# Supplementary material for: Association of vitamin D and bisphenol A levels with cardiovascular risk in an elderly Italian population: results from the InCHIANTI study
Source: GeroScience. 2024 Jun 5;46(6):6141–56. doi: 10.1007/s11357-024-01193-1 (PMC11494005; doi:10.1007/s11357-024-01193-1)
Supplement: Supplementary file 2 — Supplementary file2 (DOCX 16.7 KB) [file 11357_2024_1193_MOESM2_ESM.docx]

**Supplementary Table 1**. Colour-coded correlation matrix of different vitamin D metabolites, BPA and CV risk factors

|  | BMI | Age | 1,25(OH)D | 25(OH)D | PTH | VDBP | Creatinine | BPA | Calcium | Magnesium | Total Chol. | HDL | LDL | Triglycerides | Glucose |
| --- | --- | --- | --- | --- | --- | --- | --- | --- | --- | --- | --- | --- | --- | --- | --- |
| BMI | 1.00 |  |  |  |  |  |  |  |  |  |  |  |  |  |  |
| Age | 0.11 | 1.00 |  |  |  |  |  |  |  |  |  |  |  |  |  |
| 1,25(OH)D | **-0.13** | **-0.36** | 1.00 |  |  |  |  |  |  |  |  |  |  |  |  |
| 25(OH)D | **-0.13** | **-0.26** | **0.50** | 1.00 |  |  |  |  |  |  |  |  |  |  |  |
| PTH | **0.12** | **0.25** | **-0.22** | **-0.44** | 1.00 |  |  |  |  |  |  |  |  |  |  |
| VDBP | 0.03 | 0.05 | -0.11 | -0.02 | 0.11 | 1.00 |  |  |  |  |  |  |  |  |  |
| Creatinine | 0.03 | **-0.48** | **0.15** | 0.02 | -0.03 | -0.06 | 1.00 |  |  |  |  |  |  |  |  |
| BPA | 0.07 | **0.33** | **-0.67** | **-0.69** | **0.39** | 0.06 | **-0.14** | 1.00 |  |  |  |  |  |  |  |
| Calcium | 0.00 | 0.01 | 0.07 | **0.16** | **-0.18** | 0.06 | **-0.15** | **-0.14** | 1.00 |  |  |  |  |  |  |
| Magnesium | -0.05 | -0.02 | 0.10 | -0.04 | 0.03 | 0.07 | 0.01 | -0.02 | -0.06 | 1.00 |  |  |  |  |  |
| Total Chol. | 0.01 | -0.11 | 0.08 | 0.05 | -0.05 | 0.11 | -0.02 | -0.02 | **0.20** | 0.10 | 1.00 |  |  |  |  |
| HDL | 0.03 | **-0.14** | 0.08 | 0.07 | -0.05 | 0.10 | 0.06 | -0.06 | 0.08 | 0.09 | **0.90** | 1.00 |  |  |  |
| LDL | **-0.25** | -0.08 | **0.12** | 0.09 | -0.01 | 0.06 | -0.11 | -0.02 | **0.22** | 0.03 | **0.28** | 0.10 | 1.00 |  |  |
| Triglycerides | **0.29** | **0.16** | **-0.12** | **-0.15** | 0.02 | 0.04 | -0.10 | **0.13** | **0.20** | 0.06 | **0.34** | **0.19** | **-0.45** | 1.00 |  |
| Glucose | **0.33** | **0.15** | **-0.18** | **-0.14** | 0.02 | 0.08 | -0.05 | **0.21** | **0.19** | **-0.17** | -0.03 | -0.09 | **-0.20** | **0.29** | 1.00 |

Data are presented as Pearson correlation coefficient (*r*) and colour represents level of correlation (red, orange and light green representing good correlation, i.e. r close to 1 or -1 whereas yellow and light orange represent poor and no correlation respectively, i.e. r close to 0). BMI = body mass index, BPA = bisphenol A; CV = cardiovascular; HDL = high-density lipoprotein; LDL = low-density lipoprotein; PTH = parathyroid hormone, VDBP = vitamin D binding protein. Regression coefficients in bold text represent statistically significant correlations.
